# Facial Emotion Recognition and Eye Gaze in Attention-Deficit/Hyperactivity Disorder With and Without Comorbid Conduct Disorder

**DOI:** 10.1016/j.jaac.2018.04.016

**Published:** 2018-08

**Authors:** Jac N. Airdrie, Kate Langley, Anita Thapar, Stephanie H.M. van Goozen

**Affiliations:** a Cardiff University, Wales, UK; b MRC Centre for Neuropsychiatric Genetics and Genomics, Cardiff University, Wales, UK; c Leiden University, the Netherlands; d MRC Centre for Neuropsychiatric Genetics and Genomics, Cardiff University, Wales, UK and the Institute of Psychological Medicine and Clinical Neurosciences, Cardiff University, Wales, UK

**Keywords:** attention-deficit/hyperactivity disorder, conduct disorder, emotion recognition, eye gaze, attention

## Abstract

**Objective:**

Conduct disorder (CD) is associated with impairments in facial emotion recognition. However, CD commonly co-occurs with attention-deficit/hyperactivity disorder (ADHD); thus, it is unclear whether these impairments are explained by ADHD or by one of its core features—inattention. We explored whether emotion recognition impairments are specific to individuals with ADHD and comorbid CD while also examining the mechanisms that might explain such deficits.

**Method:**

A total of 63 male and female adolescents with ADHD (mean age = 14.2 years, age range = 11–18 years) and with (ADHD+CD) or without (ADHD) comorbid CD, and 41 typically developing controls (healthy controls [HC]; mean age = 15.5, age range = 11–18 years) performed an emotion recognition task with concurrent eye-tracking.

**Results:**

Participants with ADHD+CD were less accurate at recognizing fear and neutral faces, and more likely to confuse fear with anger than participants with ADHD alone and HC. Both ADHD subgroups fixated the eye region less than HC. Although there was a negative correlation between ADHD symptom severity and eye fixation duration, only CD severity was inversely related to emotion recognition accuracy.

**Conclusion:**

Only ADHD participants with comorbid CD showed impairments in emotion recognition, suggesting that these deficits are specific to individuals with conduct problems. However, lack of attention to the eye region of faces appears to be a characteristic of ADHD. These findings suggest that emotion recognition impairments in those with ADHD+CD are related to misinterpretation rather than poor attention, offering interesting opportunities for intervention.

Children who receive a diagnosis of conduct disorder (CD) display a persistent pattern of behavior in which the rights of others are violated and major age-appropriate social norms are breached.^1^ Not surprisingly, the behavior of these children comes with significant costs to the individual, the immediate family, and society at large, and the prognosis of such individuals is not favorable, with many becoming involved in the criminal justice system.^2^ The costs and prevalence of CD are not unique to the United Kingdom, with studies from the United States^3^ and elsewhere in Europe^4^ highlighting the increased provisions required.

One mechanism that has been found to be important in explaining the behavioral characteristics of CD is impairment in the recognition of facial expressions of emotion.^5^ Facial expressions of emotion serve important social functions.^6^ Crucially, emotional facial expressions that signal distress in others, such as fear expressions, serve as inhibitors of aggressive acts.^7^ Therefore an inability to recognize distress reduces the likelihood that aggressive acts will be inhibited, and is thought to adversely affect the development of empathy.^8^ Although fear recognition impairments in antisocial samples are found fairly consistently,^9^, ^10^, ^11^, ^12^ impairments in negative emotions more generally have also been found.^13^, ^14^

Despite evidence demonstrating emotion recognition impairments in those with CD, studies have failed to account for comorbid attention-deficit/hyperactivity disorder (ADHD). ADHD is comorbid in 30% to 50% of cases,^15^ and emotion recognition impairments have also been found in individuals with a diagnosis of ADHD.^16^, ^17^ However, one study found no difference between ADHD and typically developing controls in emotion recognition accuracy when excluding those who had comorbid CD.^18^ In addition, a recent genetic study^19^ found that impairments in fear conditioning and empathy in adolescents with ADHD were specific to those with comorbid CD. However, that study did not explore emotion recognition, and, as a result, it remains unclear whether impairments in facial emotion recognition are associated with ADHD or are found only in those with a comorbid CD diagnosis.

It is also uncertain *why* individuals with CD have emotion recognition impairments, and therefore which elements of interventions may be responsible for improvements in emotion recognition and behavior (eg, a reduction in crime^20^). Dadds *et al.*^9^, ^11^ hypothesized that a lack of attention paid to the eye region of the face, as evidenced by fewer fixations to the eye region, leads to poorer recognition. This could be especially impaired in those with ADHD, because inattention is a core symptom of this disorder. Others have proposed the existence of interpretational biases.^21^ Aggressive individuals supposedly also have a hostile attribution bias^22^ whereby neutral expressions are misattributed as hostile or threatening. Therefore, it is unclear whether emotion recognition impairments result from impairments in the attentional processing of facial expressions or a misinterpretation of the features of emotional expressions, or perhaps a combination of the two.

Given these uncertainties, the primary aim of the current study was to investigate whether impairments in emotion recognition are specific to those with comorbid CD or are a feature of ADHD itself. We also sought to examine attention and the specific errors made during emotion recognition performance to help disentangle the role of these mechanisms in emotion recognition impairments.

Adolescents with a clinical diagnosis of ADHD with or without comorbid CD and their typical developing matched controls completed an emotion recognition task while visual attention and scanning patterns were recorded via eye-tracking. We predicted that participants with ADHD and comorbid CD would exhibit impairments in negative emotion recognition and would be more inclined to identify neutral faces as angry compared to those with ADHD without CD and typically developing controls. Second, we predicted that both ADHD subgroups would demonstrate attentional problems compared to the healthy controls, but that the ADHD+CD subgroup would have a specific problem in focusing on the eye region of the face, over and above ADHD alone.

## Method

### Participants

A total of 63 adolescents (16 female and 47 male) between 11 and 18 years of age (mean = 14.2, SD = 2.09) with a clinical diagnosis of ADHD were recruited from psychiatric and pediatric clinics in Wales (UK) as part of a larger genetic study.^21^ Those with any known clinical diagnosis of schizophrenia, bipolar disorder, autism, Tourette syndrome, or with an IQ of less than 70, epilepsy, brain damage, or any genetic disorder were excluded. All participants had normal or corrected-to-normal vision. No participants were stimulant naive, but those currently being prescribed stimulant medication were asked to refrain from taking it at least 24 hours before testing. A total of 41 typically developing and healthy control participants (HC; 20 female and 21 male) between 11 and 18 years of age (mean = 15.5, SD = 2.7) were recruited from local schools. HC participants had normal or corrected-to-normal vision and were excluded if they had received an ADHD or Autism Spectrum Disorder (ASD) diagnosis.

Ethical approval was obtained from the Wales Multicentre Research Ethics Committee. Informed written consent was obtained from parents and adolescents more than 16 years of age, and written assent was obtained from adolescents less than 16 years of age.

### Measures and Materials

#### Clinical Measures

Child psychopathology in participants with ADHD was assessed using the Development and Well-Being Assessment (DAWBA) structured psychiatric research diagnostic interview, using both parents and children as informants.^23^ ADHD and CD symptom scores and diagnoses were generated from the DAWBA interview. Total symptom scores and diagnoses were computed according to *DSM-IV* criteria (the *DSM-5* had not been published at the start of the study^24^) and further verified by a trained clinician. CD symptoms were considered to be present if endorsed by either the parent or the adolescent. The internal reliability of both CD symptom (α = .78) and ADHD symptom severity (α = .88) was high.

ADHD group membership was based on whether or not participants met *DSM-IV* diagnostic criteria for conduct disorder; this resulted in an ADHD only (n = 36) subgroup and an ADHD with comorbid CD (ADHD+CD; n = 27) subgroup.

CU traits were measured using the Youth Psychopathic traits Inventory (YPI),^25^ the validity of which has been demonstrated.^26^, ^27^, ^28^ The internal consistency of the CU subscale (α = .80) and YPI overall (α = .94) was good. To be able to compare across ADHD and control participants, CD symptoms were assessed using the parent and child Strengths and Difficulties Questionnaire (SDQ; α = .82); the SDQ has high specificity and sensitivity in identifying CD symptomatology.^29^

In those with ADHD, estimated IQ was obtained via two subtests of the Wechsler Abbreviated Scale of Intelligence.^30^

SES was estimated using the UK’s Office of National Statistics estimates of average house-hold weekly income based on participants’ postcodes (low = £0–520; middle = £521–670; high = £671+).

#### Emotion Recognition

Emotion recognition was examined using the Facial Emotion Recognition task,^31^ consisting of 60 faces from the Ekman and Friesen facial affect battery,^32^ representing four basic emotions (happiness, anger, fear, sadness) and neutral. Each emotion was morphed with its corresponding neutral expression to create a 50% and 75% intensity expression; 50% represents a central point between the facial feature configuration of a neutral expression and a 100% basic emotion, whereas 75% represents the halfway point between the 50% facial configuration and 100% configuration. An equal number of male and female target faces appeared, and slides contained an equal number of each emotion presented at each intensity. Each trial presented a target image along with numbered options of 1 to 5, representing, “Happy, “Sad,” “Fear,” “Anger,” and “Neutral.” The Facial Emotion Recognition task has good reliability (α = .83) and has been used in previous studies with adolescents.^20^, ^31^

#### Eye-Tracking

Participants were positioned 60 to 65 cm from a laptop screen, and a 9-point calibration was performed. The quality of calibration was checked and repeated as required. Calibration was followed immediately by facial stimuli. Eye-movements were recorded with a portable Tobii X2-60 compact eye-tracker sampling at 60 Hz with a screen resolution of 1920 × 1080. This equipment is robust to changes in head position, removing the need for a chin rest. An I-VT fixation filter with a minimum fixation criterion of 60 milliseconds sampled average raw data of both eyes to produce information on eye position and duration. Eye-gaze validity was checked using a sample rate percentage that gives an estimate of the quality of eye-tracking in a recording by providing a percentage score of successfully recorded data.

### Procedure

Participants with ADHD and ADHD+CD were tested in a dimly lit laboratory room in a university clinic. HC participants were tested in a dimly lit room at school. Following eye-tracker calibration, each face was presented using a set of three slides. First, a noise screen was used to prevent visual carryover effects from the previous trial; second, a fixation cross-controlled starting eye position; and third, the face stimulus was presented. Noise and fixation screens were presented for 1 second each. The face stimulus had no time constraint, lasting for as long as it took to select an emotion.

### Data Analyses

#### Emotion Recognition Accuracy

Percent correct recognition and percent incorrect emotion selected for each emotion and each intensity level were calculated.

#### Eye-Tracking

Tobii Studio was used to analyze eye gaze. Areas of interest (AOIs) were created around the eyes, mouth, and face as a whole; one AOI was created around the emotion options and another around the entire screen. Percentage of dwell time to the eyes was calculated by summing all fixations to the eye AOI divided by the total duration of time spent looking at the face AOI. We also analyzed time to first fixation on the eye (TFF). Values more than 2 standard deviations above the mean and trials with values of zero were excluded from the TFF analyses.

Analyses were carried out using SPSS version 20 (SPSS Inc., Chicago, IL). Differences in demographic and clinical characteristics between groups were analyzed with one-way analyses of variance (ANOVAs) for continuous variables, with Student *t* tests used to determine specific differences between groups and χ^2^ tests for binary variables. Pearson (Spearman rho where appropriate) correlations were used to examine relationships between demographic and clinical characteristics with recognition and eye-gaze measures. Mixed-model ANOVAs (or analyses of covariance where demographics differed between groups and were independently related to outcome measures), with group (ADHD+CD, ADHD, HC) as a between-subjects factor and emotion (fear, happy, sad, angry) and intensity (50% and 75%) as within-subjects factors were used for the analysis of emotion recognition and eye-tracking measures. Where intensity was not found to interact with group, the two intensities were merged, and ANOVAs were rerun without intensity as a factor and the addition of neutral to the emotion factor. To limit the number of multiple comparisons, statistical analysis of confusion errors, using one-way ANOVAs, were limited to emotions where a significant group difference was found. Where the assumption of sphericity was violated, Greenhouse−Geisser corrections were used. Where follow-up tests were required, Bonferroni corrections were used. Effect sizes were calculated as partial eta squared (η_p_^2^; small ≥ 0.01, medium ≥ 0.06, large ≥ 0.14).^33^

## Results

Table 1 presents demographic and clinical characteristics of the study groups. The groups differed in gender (χ^2^(2) = 7.4, *p* = .025) and age (*F*_2,103_ = 4.94, *p* = .009). Emotion recognition accuracy did not differ between genders (*F*_1,103_ = 2.57, *p* = .11); however, the relationship between age and emotion recognition approached significance (*r*_104_ = 0.054). ANOVAs were therefore conducted both with and without including age as a covariate. Both analyses resulted in the same pattern of results in terms of direction and significance, so results are reported below without using age as a covariate.

**Table 1:** Demographic and Clinical Characteristics of Sample

Characteristic	ADHD+CD (n = 27)	ADHD (n = 36)	HC (n = 41)	*p*	Post Hoc
Age	13.7 (2.18)	14.6 (2.02)	15.51 (2.68)	.009	ADHD+CD < HC
IQ_WASI_	80.5 (13.72)	86.17 (16.93)	NA	.17	
% Female	17.9	30.6	48.8	.03	
SES				.46	
% Low	60.7	41.7	43.6		
% Medium	28.6	44.4	35.9		
% High	10.7	13.9	20.5		
CD_DAWBA_	6.44 (2.34)	1.0 (1.03)	NA	<.001	
CD_SDQ_	5.26 (2.25)	2.74 (1.44)	1.81 (1.38)	<.001	ADHD+CD > ADHD ADHD+CD, ADHD > HC
ADHD_DAWBA_	14.15 (3.61)	11.89 (4.6)	NA	.042	
CU_YPI_	36.59 (8.03)	30.03 (7.89)	32.04 (5.68)	.002	ADHD, HC < ADHD+CD

Note: Means are presented with standard deviations in parentheses (except where indicted otherwise). Both ADHD_DAWBA_ and CD_DAWBA_ represent number of symptoms and are restricted to the ADHD groups. IQ is also restricted to ADHD groups. CD_SDQ_ is CD score as measured by SDQ. CU_YPI_ is CU subscale for YPI. ADHD = attention-deficit/hyperactivity disorder; ADHD_DAWBA_ = ADHD symptoms measured by the DAWBA; CD = conduct disorder; CD_DAWBA_ = CD symptoms measured by the DAWBA; CD_SDQ_ = conduct score measured by SDQ: CU = callous unemotional; CU_YPI,_ = callous unemotional traits measured by Youth Psychopathy Inventory; DAWBA = Development and Well-Being Assessment; HC = healthy controls; IQ_WASI_ = intelligence quotient (two-subtest Wechsler Abbreviated Scale of Intelligence); NA = not applicable; SDQ = Strengths and Difficulties Questionnaire; SES = socioeconomic status; YPI = Youth Psychopathy Inventory.

The ADHD+CD group had (as expected by definition) significantly more CD symptoms than the ADHD alone group and controls (all *p* < .001) and more ADHD symptoms than ADHD alone (*p* = .042). There was a significant group difference in CU traits (*p* = .02), with ADHD+CD having a higher CU score than ADHD (*p* = .002) and controls (*p* .035), whereas ADHD and controls did not differ (*p* = .66).

### Facial Emotion Recognition

There was a main effect of intensity (*F*_1,101_ = 382.4, *p* < .001, ηp^2^ = 0.79), with more accurate reports for higher intensity emotions (mean = 78.2, SD = 10.77 versus mean = 61.06, SD = 12.52). There was no interaction between intensity and group (*F*_2,101_ = .443, *p =* .64, ηp^2^ = .009). Scores were therefore averaged across intensities, and analyses were rerun without intensity as a factor.

**Figure 1:**
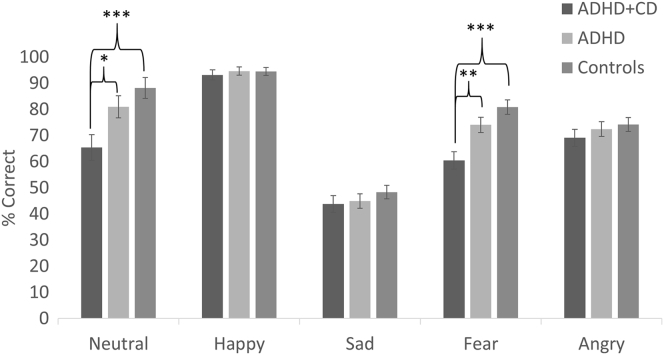
Percentage of Correct Responses to Each Emotion for Each Group ***Note**: Error bars denote ±1 standard error. ADHD = attention-deficit/hyperactivity disorder; CD = conduct disorder.* ∗p *< .05, ∗∗*p *< .01, ∗∗∗*p *< .001*.

Recognition accuracy scores are presented in Figure 1. There was a main effect of emotion (*F*_2.76,278.9_ = 110.2, *p* < .001, ηp^2^ = 0.52), with highest accuracy scores to happy faces (mean = 94.23 SD = 9.65), followed by neutral (mean = 79.81, SD = 26.75), fear (mean = 73.23, SD = 19.14), angry (mean = 72.28, SD = 16.97), and sad (mean = 45.99, SD = 16.63). There was also a main effect of group (*F*_2,101_ = 9.6, *p* < .001, ηp^2^ = 0.16), and a significant interaction between group and emotion (*F*_5.52,278.9_ = 2.98, *p* = .01, ηp^2^ = 0.056). Groups differed in fear (*p* < .001) and neutral (*p* = .002) recognition but not in other emotions (all *p* > .05). ADHD+CD were less accurate in recognition of fear and neutral expressions than ADHD (*p* < .003 and *p* =.018 respectively) and HC (*p* < .001 and *p* < .001 respectively), whereas there was no difference in fear or neutral recognition between ADHD and HC (*p* > .05).

Because most studies focus on recognition accuracy in males only, we conducted a sensitivity analysis restricted to males and found the same pattern of results, with a significant interaction between emotion and group (*F*_5.06,164.3_ = 2.71, *p* = .02, ηp^2^ = 0.08), with ADHD +CD participants showing less accurate fear and neutral recognition accuracy than ADHD and controls (all *p* < .05) and no difference between ADHD and HC (*p* > .05). Although the small number of females precluded any statistical analysis of the performance of females only, the direction of results in females was the same as that of males and the sample as a whole.

### Confusion Matrix

#### Fear

Patterns of errors made by the groups are presented in Table 2. There was a significant main effect of group in incorrectly identifying fear faces as sad (*F*_2,103_ = 6.39, *p* = .002, ηp^2^ = 0.11). ADHD+CD made this error more than HC (*p* < .002). There was no difference between ADHD+CD and ADHD. There was also a significant group difference in tendency to misinterpret fear faces as angry (F_2,103_ = 5.95, *p* = .004, ηp^2^ = 0.11); ADHD+CD made this error more than ADHD (*p* = .04) and HC (*p* = .003), with no difference between ADHD and HC (*p* > .05).

**Table 2:** Confusion Matrices Depicting the Mean Percentage That Participants Selected Each Option for Each Presented Emotion

		Emotion Selected														
		Neutral			Happy			Sad			Fear			Angry		
		1	2	3	1	2	3	1	2	3	1	2	3	1	2	3
Emotion presented	Neutral	**65.4**^a^^,^^b^	**81.0**^a^	**88.21**^b^	11.1^a^^,^^b^	2.8^a^	3.7^b^	12.4^a^	5.6	1.2^a^	3.7	3.7	0.0	7.4	6.9	6.9
	Happy	4.6	3.4	4.8	**93.2**	**94.7**	**94.2**	0.93	0.46	0.41	0.93	.93	0.0	0.31	0	.20
	Sad	37.0	38.9	41.36	4.6	2.3	1.2	**43.8**	**44.9**	**48.4**	8.0	9.3	5.9	6.5	3.7	3.3
	Fear	12.7	8.1	8.9	1.5	1.6	0.81	13.3^b^	10.0	5.5^b^	**60.5**^a^^,^^b^	**74.1**^a^	**80.9**^b^	11.4^a^^,^^b^	5.6^a^	3.9^b^
	Angry	10.5	10.9	17.1	0.31	1.2	0.61	9.3	3.7	3.1	10.5	10.9	5.1	**69.1**	**72.5**	**74.2**

Note: Boldface values depict correct responses. 1 = ADHD+CD; 2 = ADHD; 3 = HC. ADHD = attention-deficit/hyperactivity disorder; CD = conduct disorder; HC = healthy control.

a Significant difference between ADHD+CD and ADHD groups.

b Significant difference between ADHD+CD and control groups.

#### Neutral

There was a significant group difference in tendency to judge neutral faces as happy (*F*_2,103_ = 5.17, *p* = .007, ηp^2^ = 0.09). ADHD+CD was more likely to make this error than both ADHD and HC (all *p* < .01), whereas there was no difference between ADHD and HC (*p* > .05). There was also a group difference in the propensity to judge neutral faces as sad (*F*_2,103_ = 5.24, *p* = .007, ηp^2^ = 0.09), with pairwise comparisons indicating that ADHD+CD made this error more than HC (*p* <.01*)*; the differences between ADHD+CD and ADHD approached significance (*p* = .057). There were no group difference in the tendency to misinterpret neutral faces as angry (*F*_2,103_ = .18, *p* = .98, ηp^2^ < 0.001), or fearful (*F*_2,103_, *p* = .72, ηp^2^ =0.05).

### Eye-Tracking

Examination of participants’ eye-gaze validity led to the exclusion of three ADHD+CD participants, three ADHD participants, and one HC participant.

#### Proportion of Time Spent Looking at the Eyes

A three-way ANOVA with emotion and intensity as within-subjects factors and group as a between-subjects factor revealed a main effect of intensity (*F*_1, 94_ = 5.22, *p* = .025, ηp^2^ = 0.053), with participants spending more time focusing on the eyes for higher-intensity (mean = 56.07, SD = 17.48) compared to lower-intensity (mean = 54.71, SD = 17.48) faces. There was no interaction between emotion and intensity (*F*_3,282_ = 0.57, *p* = .64, ηp^2^ = 0.006), or between intensity and group (*F*_2,94_ = 0.60, *p* = .55, ηp^2^ = 0.013). Scores were therefore averaged across emotion intensities.

**Figure 2:**
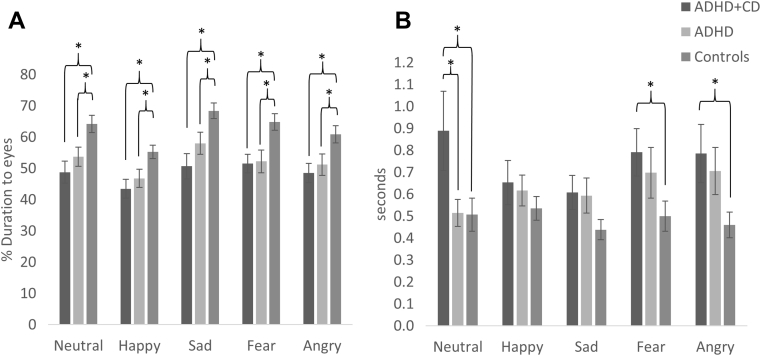
Fixation on Eye Regions. (A) Percentage of Fixations on the Eye Region Out of All Fixations on the Face. (B) Mean Time to First Fixation on Eye Region ***Note**: Error bars denote ±1 standard error. ADHD = attention-deficit/hyperactivity disorder; CD = conduct disorder.* *∗*p *< .05, ∗∗*p *< .01, ∗∗∗*p *< .001.*

A main effect of emotion was found (*F*_3.54,332.86_ = 26.72, *p* < .001, ηp^2^ = 0.22) (Figure 2A*)*. Participants focused most on the eyes for sad faces (mean = 60.49, SD = 19.65), followed by fear (mean = 57.23, SD = 18.89), neutral (mean = 56.79, SD = 18.54), angry (mean = 54.47, SD = 18.07), and happy (mean = 49.4, SD = 15.71) faces. There was also a main effect of group (*F*_2,94_ = 6.81, *p* = .002, ηp^2^ = .13), with both ADHD+CD and ADHD groups spending less time focusing on the eyes than HC (all *p* < .05), whereas there was no difference between ADHD+CD and ADHD (*p* > .05). There was no interaction between emotion and group (*F*_7.08,332.86_ = 1.33, *p* = .24, ηp^2^ = 0.03). This pattern of findings did not change when we included time taken to identify the faces as a covariate.

#### Time to First Fixation on Eye Region

After removing participants with a mean TFF of zero, 22 ADHD+CD, 31 ADHD, and 35 HC participants remained.

There was a main effect of group (*F*_2,85_ = 3.25, *p* = .04, ηp^2^ = 0.07), but no main effect of emotion (*F*^3.25,275.86^ = 1.76, *p* = .15, ηp^2^ = 0.02), and no interaction between group and emotion (*F*_6.49,275.86_ = 1.82, *p* = .089, ηp^2^ = 0.04). ADHD+CD were slower to fixate to the eyes than HC (*p* < .05), but there was no difference between ADHD+CD and ADHD or between ADHD and HC (all *p* > .05). Pairwise comparisons indicated that ADHD+CD were slower to fixate the eye region than HC for fear and angry faces (all *p* < .05), whereas ADHD+CD was slower than both ADHD and HC for neutral faces (all *p* < .05). There were no differences between groups for the other emotions (all *p* > .05).

### Relationships Among Clinical Characteristics, Emotion Recognition, and Fixation to Eyes

Table 3 presents correlations among clinical characteristics, emotion recognition, and fixation duration for those emotions for which there was a significant group difference. A negative relationship was found between severity of CD and emotion recognition in individuals with ADHD. Although CU trait scores were significantly negatively related to emotion recognition accuracy overall and to fear recognition specifically, these relationships were weaker than the corresponding relationships with CD severity. CD symptoms did not relate to time spent looking at the eyes. ADHD symptom severity was negatively related to percentage of time spent looking at the eyes but was unrelated to emotion recognition accuracy. Across the sample as a whole, there was a positive relationship between emotion recognition and percentage of time spent looking at the eyes.

**Table 3:** Relationships Among Clinical Variables, Emotion Recognition Accuracy, and Percentage of Fixation Duration to the Eyes

	1		2		3		4		5		6		7		8		9	
	n	*r*	n	*r*	n	*r*	n	*r*	n	*r*	n	*r*	n	*r*	n	*r*	n	*r*
CD_DAWBA_	–	–																
CDSDQ	61	0.53∗∗∗	–	–														
CUYPI	61	0.51∗∗∗	102	0.37∗∗∗	–	–												
ADHD_DAWBA_	61	0.33∗	60	0.22∗	60	0.173	–	–										
Emotion accuracy overall	62	–0.23	103	–0.29∗∗	103	–0.19∗	61	–0.15	–	–								
Fear accuracy	62	–0.27∗	103	–0.29∗∗	103	–0.20∗	61	–0.19	0.104	0.67∗∗∗	–	–						
Neutral accuracy	62	–0.24	103	–0.24∗	103	–0.08	61	–0.23	104	0.48∗∗∗	104	0.28∗∗	–	–				
% Fear eyes	56	–0.11	97	–0.25∗	96	–0.19	55	–0.35∗	97	0.37∗∗∗	97	0.34∗∗	97	0.35∗∗∗	–	–		
% Neutral eyes	56	–0.12	97	–0.26∗	96	–0.16	55	–0.45∗∗	97	0.32∗∗	97	0.28∗∗	97	0.28∗∗	97	0.81∗∗∗	–	–
% Eyes all	56	–0.17	97	–0.26∗	96	–0.19	55	–0.41∗∗	97	0.31∗∗	97	0.31∗∗	97	0.32∗∗	97	0.95∗∗∗	97	0.91∗∗∗

Note: Correlations involving CD_DAWBA_ or ADHD_DAWBA_ only include participants in either of the ADHD groups. ADHD = attention-deficit/hyperactivity disorder; ADHD_DAWBA_ = ADHD symptoms measured by the DAWBA; CD = conduct disorder; CD_DAWBA_ = CD symptoms measured by the DAWBA; CD_SDQ_ = conduct score measured by the SDQ; CU_YPI_ = callous unemotional traits measured by Youth Psychopathy Inventory; DAWBA = Development and Well-Being Assessment; n = sample size; r = Pearson r; SDQ = Strengths and Difficulties Questionnaire; % = percentage of fixation duration.

∗ *p* < .05, ∗∗*p* < .01, ∗∗∗*p* < .001.

## Discussion

We compared emotion recognition between ADHD+CD, ADHD alone, and healthy control participants to ascertain whether emotion recognition impairments were evident in individuals with a diagnosis of ADHD. We found support for the hypothesis that these impairments are specific to those ADHD participants with additional CD, with evidence of specific impairments in the recognition of fear and neutral faces. Although a null result by itself cannot be taken as evidence of equivalent performance, when the absence of significant differences between ADHD and control participants is seen in the context of previous studies of individuals with conduct disorder, this supports the idea that emotion recognition deficits are specific to those with additional CD. Our findings are in line with studies showing deficits in fear recognition in antisocial populations,^9^, ^11^, ^12^ but are not consistent with some studies of antisocial or CD samples in which additional impairments in sadness^20^, ^31^ or anger were found.^34^, ^35^ Differences in sample composition and/or the specific design of the emotion recognition tasks may help to explain these inconsistencies.

The absence of a difference in emotion recognition performance between ADHD alone and typically developing controls is inconsistent with studies in which recognition deficits in ADHD were found.^16^, ^17^ However, participants in a study by Pelc *et al.*^16^ were considerably younger than the present sample (7–12 years), and the study protocol of Singh *et al.*^17^ did not include a pure measure of facial emotion recognition. Furthermore, our findings are consistent with other studies in which evidence of emotion (recognition) impairments was limited to those with comorbid CD.^18^, ^19^, ^36^

The study’s second aim was to gain a clearer understanding of the mechanisms involved in emotion recognition impairments. Although it has been argued that individuals with aggressive behavior exhibit a hostile attribution bias,^21^ participants in the ADHD+CD group were not more prone to confuse neutral with anger. Because evidence for the hostile attribution bias was originally found in a study^21^ examining the proposed intention of a character in a situational context, it is possible that the bias is specific to attributions of intent. However, participants with ADHD+CD were more likely to misinterpret fear as anger. If fear were misinterpreted as anger in a confrontational situation, an inhibitory cue would not be available to an (already) aggressive individual, and this might lead to more aggression and violence. We also found that participants with ADHD+CD had a tendency to confuse fear with sadness, which should lead to an inhibited response but might nevertheless be a less potent inhibitor. In any case, ADHD+CD participants appear to have a general difficulty in the interpretation of fearful features. Interestingly, we found an inverse relationship between CD severity and emotion recognition accuracy, providing further evidence that emotion recognition in general, and fear recognition in particular, are problems for those with ADHD and comorbid CD.

To our knowledge, this is the first study to examine eye-gaze in ADHD participants while they were performing an emotion recognition task. The finding that participants with ADHD looked less at the eye region of a face is in line with a recent study that found that those with ADHD were less distracted by the eye-gaze of distractor faces while performing a word classification task compared to controls, suggesting that the attention of participants with ADHD was captured less by eye-gaze.^37^ The fact that participants in both ADHD groups looked at the eyes less for all emotions compared to healthy controls suggests that lack of attention to the eye region is a problem in ADHD generally, rather than a specific problem for those with comorbid CD, and that emotion recognition deficits in those with comorbid CD are not due to a lack of attention to the eye region. This might seem to be inconsistent with a study in which it was that found directing participants to look toward the eyes eliminated fear recognition impairments.^9^ However, participants in that study were typically developing undergraduates divided into those with high or low CU traits, and therefore a very different sample from one used in the current study. Also relevant here is the fact that CD symptom severity was more strongly related to emotion recognition impairments than were CU traits.

Our findings are important for the design of interventions targeting emotion recognition deficits as a cognitive mechanism underlying CD, including in those with ADHD. In a juvenile offender emotion intervention study,^20^ participants were not only taught to pay attention to salient features of the face but were also given hints to assist with the interpretation of features. The current findings suggest that the improvements observed in that study may have been due to help with the interpretation of features rather than redirecting attention to relevant parts of the face.

In support of the finding that neglect of the eye region is a problem for individuals with ADHD generally, we found a stronger negative correlation between ADHD severity and time spent looking at the eyes than with CD severity. This suggests that it is ADHD symptomatology rather than CD symptomatology that is driving this attentional problem.

We also examined time to first fixation on the eye region. Although participants with ADHD+CD were no different from those with ADHD alone, they were slower to engage attention to this region than were controls. This suggests that it takes longer for individuals with comorbid CD to engage with this important area of the face. If our paradigm had been time limited, the ADHD+CD group might have shown stronger recognition impairments due to not having the opportunity to process this region.

The findings of the current study should be interpreted in light of some limitations. First, we used a sample of adolescents with ADHD, categorized them into those with or without CD, and compared them to typical developing healthy controls matched for socioeconomic status to determine whether emotion recognition impairment was a general problem for participants with ADHD or evident only in those with ADHD with additional CD. The study would be strengthened by the inclusion of a sample of adolescents with CD alone. Although previous studies have shown that adolescents with CD exhibit impairments in facial emotion recognition compared to controls,^38^ in the absence of a CD-alone group, we cannot be sure that such emotion recognition impairments are specific to the combination of ADHD and CD. It would be informative to know whether individuals who have combined ADHD and CD are more impaired in emotion recognition than those with CD alone.

Second, practical limitations prevented the collection of IQ data in controls during school hours. It seems very likely that the healthy controls’ IQ scores would have been higher than those of the ADHD groups, and it is, of course, plausible that emotion recognition performance is correlated with IQ. However, IQ differences cannot account for the finding that healthy controls performed better than the ADHD+CD subgroup but not the ADHD subgroup, and yet the two ADHD subgroups did not differ in IQ this strongly. This makes it implausible that group differences in emotion recognition resulted from IQ differences.

Third, one could argue that our paradigm lacks ecological validity. Participants had unlimited time to process the faces and to respond. This procedure was adopted to reduce the need for participants to rely on memory. However, real-life scenarios are typically time limited. It is therefore possible that participants’ emotion recognition accuracy was overestimated and that larger differences between groups would have emerged if less time had been given.

Fourth, the effect sizes of the emotion recognition differences between groups were smaller than the differences in recognition accuracy among emotion categories, although they were still in the medium-to-large range.^33^ Therefore the clinical significance of these impairments remains unclear. Nevertheless, an intervention study of young offenders has shown that improving emotion recognition led to a reduction in the severity of subsequently committed crimes,^20^ which suggests that the ability to recognize emotions has important consequences for behavior. Future studies should explore whether improving emotion recognition in those with comorbid ADHD and CD leads to a reduction in CD symptom severity over time.

Finally, the eye-tracking software that we used restricted us to exploring overall eye-gaze patterns, regardless of whether the response given was correct or incorrect, and future studies should explore the eye-gaze in incorrect trials to determine whether the pattern of eye-gaze reflects the selected incorrect emotion. Aviezer *et al.*^39^ showed that the context in which an emotional face was displayed affected eye-gaze patterns, with areas of the face important in conveying information about the emotion expected in the given context being looked at more. It is possible that individuals with ADHD and CD engage in eye-gaze patterns that are consistent with the emotion that they expect; if their expectations are wrong, they would fail to attend to the facial features that would provide information relevant to making the correct choice.

Developing and providing early interventions that prevent and reduce problem behavior in children with CD are crucial. Although parenting interventions are well established, there is growing appreciation that interventions that target underlying neurocognitive mechanisms might also be important. Using a clinical sample of adolescents with ADHD divided into those with and without comorbid CD, and comparing these with a sample of typically developing controls, we found that recognition of fear and neutral facial expressions was specifically impaired in individuals with ADHD and comorbid CD. We also found that a lack of attention to the eye region of faces was evident in both ADHD groups, rather than being limited to participants with CD. Our findings add to discussions about the hostile attribution bias theory, in that there was no evidence among those with ADHD (with or without CD) of an increased tendency to interpret neutral faces as angry. Instead, there was evidence that ADHD+CD participants misinterpreted fear as anger, which could exacerbate behavioral problems.^7^ The fact that individuals with ADHD and comorbid CD have problems in recognizing specific emotions is relevant for interventions seeking to reduce their conduct problems by improving emotion recognition. Such interventions should aim to improve the interpretation of facial configurations typical of fear in individuals who are at risk for future conduct problems.
